# Influence of Glutamate Neurotransmission Genes on the Outcomes of Antipsychotic Treatments

**DOI:** 10.1055/a-2603-0871

**Published:** 2025-06-17

**Authors:** Marc Cendrós, Rosa Catalán, Mercè Torra, Rafael Penadés, Alexandre González-Rodríguez, Mercè Brunet, Josefina Perez-Blanco, Natalia Cullell, Alexandre Serra-Llovich, Marta H. Hernandez, María J. Arranz

**Affiliations:** 1 Eugenomic S.L., Barcelona, Spain; 2 Fundació Docència i Recerca Mútua Terrassa Terrassa, Spain; 3 Centro de Investigación Biomédica en Red de Salud Mental (CIBERSAM), Institut de Recerca Biomèdica Sant Pau (IIB-Sant Pau), Barcelona, Spain; 4 Barcelona Clinic Schizophrenia Unit (BCSU), Neurosciences Institute, Hospital Clínic of Barcelona, University of Barcelona, IDIBAPS, Barcelona, Spain; 5 Pharmacology & Toxicology Unit, Dept. Biochemistry & Molecular Genetics, Hospital Clínic of Barcelona, Barcelona, Spain; 6 Dept. Mental Health, Mutua Terrassa University Hospital, Fundació Docència i Recerca Mutua Terrassa, University of Barcelona, CIBERSAM, Barcelona, Terrassa, Spain; 7 Dept. Psychiatry, Hospital de la Santa Creu i Sant Pau, Barcelona, Spain; 8 School of Health Sciences Blanquerna, University Ramon Llull, Barcelona, Spain

**Keywords:** pharmacogenetics, psychiatry, schizophrenia, glutamate, antipsychotics

## Abstract

**Introduction:**

Traditionally, the aetiology of schizophrenia has been attributed to dopaminergic neurotransmission, but more recent information points to the role of glutamate pathways. Glutamatergic involvement in schizophrenia might be extensible to drug response. The aim of the study was to explore whether the variation in glutamate receptors, transporters and metabolism can influence the outcome of drug treatments.

**Methods:**

A total of 45 polymorphisms in the genes GRIN1, GRIN2A, GRIN2B, GRIN3A, GRIA1, GRIK2, GRM2, GRM3, GRM5, GRM8, SLC1A1, SLC1A3 and GAD1 were genotyped in 258 patients with schizophrenia. Efficacy and side effects were evaluated with the Positive and Negative Symptoms Scale and the UKU scale, respectively, at baseline and after 12 weeks.

**Results:**

The analysis revealed associations between outcomes, including response and adverse effects and genetic variants in several genes (GAD1, GRIA1, GRIN2A, GRIN3A, GRIK2, GRM2, GRM5, GRM8 and SLC1A3). An association of rs1864205 in GRIA1 with autonomic side effects bordered statistical significance after correction for multiple comparisons.

**Discussion:**

Our results suggest that genetic variation in glutamatergic pathways can influence the efficacy and safety of antipsychotic drugs.

## Introduction

Schizophrenia is a common disorder with an estimated prevalence of 1% of the worldwide population 1 . Its main clinical manifestations are positive symptoms, defined as an excess of normal cognitive processes, including hallucinations and delusions, and negative symptoms, which are a degradation of normal behaviour such as apathy, lack of emotion, initiative and self-care. In addition to this classical biaxial classification, the disorder also presents additional symptoms, remarkably a decline in cognitive function such as impaired processing speed, working memory or executive functions. Schizophrenia can have a negative impact on interpersonal relations, as well as educational and professional functioning. Moreover, affected patients are subject to stigmatization and discrimination on top of the disabilities inherent to the condition.

Dopaminergic overactivation has been the standing paradigm regarding the aetiology of schizophrenia 2 . This hypothesis was supported by the fact that certain dopaminergic drugs, such as amphetamine, can trigger episodes very similar to positive symptoms in exposed, otherwise healthy, people. Varying degrees of antagonism on the dopamine receptor D2 (DRD2) is a common characteristic of antipsychotics 3 4 , although newer drugs also rely on other mechanisms. However, this dopaminergic paradigm does not explain psychotic manifestations other than positive symptoms. More recently, a different idea has taken form in the concept of glutamate dysregulation. Ketamine, an antiglutamatergic drug, can trigger both positive and negative symptoms, and this glutamatergic paradigm could be a better explanation of the physiological changes of the schizophrenic brain 5 6 .

Genetic variants in several genes involved in glutamatergic transmission and homeostasis have been associated with schizophrenia and pharmacotherapy response in previous studies. Alterations in the regulation and expression of genes encoding N-methyl-D-aspartate (NMDA) receptor subunits have been observed in patients with schizophrenia 7 8 9 13 . Genetic variation in Glutamate Receptor, Ionotropic, NMDA-activated 2 A and 2B (GRIN2A and GRIN2B) subunits has been investigated regarding the risk of schizophrenia and response to clozapine 10 11 12 13 14 . The gene encoding glutamate receptor, ionotropic, AMPA-activated 1 (GRIA1) is suspected to influence the risk of schizophrenia, as it is located in 5q33, a region that has been associated with schizophrenia in genome-wide association studies (GWAS) 15 . Several genetic polymorphisms within this gene have been previously linked to the disorder and with treatment response 15 16 17 . The gene encoding glutamate receptor, ionotropic, kainate-activated 2 (GRIK2) is located in a genomic region associated with schizophrenia 18 . Expression of GRIK2 is reduced in people with schizophrenia 19 . Metabotropic receptors are also promising candidates for modifiers of the disorder. Schizophrenia patients have been found to have decreased methylation of the glutamate receptor, metabotropic 2 and 5 (GRM2 and GRM5) 20 . Genetic variation in GRM3 has been associated with positive and negative symptoms 21 . Regarding GRM8, several polymorphisms in this gene have been associated with schizophrenia in different studies 22 23 . Regarding transporters, genetic polymorphisms in the solute carrier 1A1 (SLC1A1) has been associated with the risk of schizophrenia 24 25 26 , whereas genetic variation in SLC1A3 has been associated with cognitive decline in patients with schizophrenia 27 28 . Patients with schizophrenia also show altered expression of SLC1A1 in their prefrontal cortex 29 . Glutamate decarboxylase 1(GAD1) is an important regulator of glutamate, and some common polymorphisms have been found to be more represented in schizophrenia patients 30 . Some preliminary information also link this gene to treatment-resistant schizophrenia 31 .

Polymorphisms affecting glutamatergic transmission may lead to qualitatively different characteristics of the disease that could confer differential response to medication, regarding either efficacy or side effects. Furthermore, the exact mechanism of action of atypical antipsychotics is not totally understood, and it is possible that they might interact with glutamatergic receptors. Limited evidence suggests that antipsychotic treatments can regulate the expression of glutamate transporters 32 . Therefore, there are multiple mechanisms through which genetic polymorphisms in glutamatergic genes can influence the outcome of currently used antipsychotics. Involvement of glutamatergic genes in schizophrenia has been seen in GWAS, such as GRM7, which has been reported to influence positive symptoms 33 . The GWAS approach is a strategy with the strong benefit that it does not require a prior hypothesis, and therefore can unveil genetic effects even when the current understanding of the pathology and its mechanisms is incomplete, as well as discover associations outside of the suspected mechanisms. However, GWAS analyse thousands of positions and require very strict statistical corrections to account for multiple comparisons. As a result, true associations can fail to reach genome-wide significance, and some known associations in candidate genes have not shown up in GWAS results 34 . This caveat can be dealt with candidate genes studies, which can find these associations with much smaller sample sizes, at the cost of missing the effect of polymorphisms outside the hypothesis.

The aim of this study was to explore the influence of genetic variation in genes encoding glutamate receptors, or influencing glutamate neurotransmission, on the response or safety of antipsychotic treatments. To achieve this objective, we investigated a selection of polymorphisms in glutamate-related genes in a cohort with detailed information on the outcome of antipsychotic treatments.

## Experimental procedures

### Sample

Patients were recruited in the mental health wards of three hospitals (Hospital Clínic and Hospital de la Santa Creu i Sant Pau, Barcelona; Hospital Universitario, Granada) and were mostly outpatients. Patients whose treatment was guided by a pharmacogenetic test 35 were excluded to avoid confounding by the intervention. Only the patients whose treatment was not changed according to pharmacogenetic information were included in the present study. Two hundred fifty-eight patients met these criteria and thus were eligible to be included in the study. The sample included patients of Caucasian ethnicity with disorders within the schizophrenia spectrum (DSM-V). Fifty-eight percent of patients were male, with an average age of 45.5 years (SD=13.76). All patients were on monotherapy with different antipsychotics: clozapine (44%), risperidone (12%), aripiprazole (7%), olanzapine (13%), quetiapine (6%), paliperidone (12%), other antipsychotics (5%) (see Table 1 ). A sample size of 120 was required to detect associations of strong effects (F=0.35), assuming an allelic frequency of 0.05 at a desired statistical power of 90%. This sample has between 80 and 99% statistical power to detect associations of moderate or large effect size assuming a minor allele frequency of 0.05 (F ^2^ =0.15–0.35, respectively, calculated with G*Power v.3.1.9.4). The patients were not taking any other drug for the duration of the study other than the antipsychotic of choice, with the sole exception of lorazepam, which was given to 17% of the patients with sleeping difficulties. This percentage was lower (6%) among patients using clozapine or olanzapine. Doses of 1 to 1.5 mg/day were used, and administration was as needed to control insomnia. All participants gave their written informed consent for the study, which was carried out in accordance with the provisions of the Helsinki Declaration. The study was approved by the scientific research ethics committee of Hospital Clinic de Barcelona (Reg. 2011/6635).

**Table:** **Table 1** Demographics.

Sex	
Males	58%
Age	
Mean	45.5
SD ^1^	13.76
Disease statistics (name WIP)	
Average initial PANSS	94.34
Average final PANSS	68.4
Average initial UKUs	7.54
Average final UKUs	3.51
Treatment	
Clozapine	44%
Olanzapine	13%
Risperidone	12%
Paliperidone	12%
Aripiprazole	7%
Quetiapine	6%
Other	5%
Average dose (ol.eq.) ^2^	11.1
Diagnostic	
Schizophrenia	76%
Delusional disorder	21%
Schizoafective disorder	3%
Total N	258

1 (SD) – Standard Deviation, 2 (ol.eq.) – olanzapine equivalent. PANSS: Positive and Negative Symptoms Scale; UKU: Udvalg for Kliniske Undersøgelser.

### Biochemical parameters

Plasma concentrations of the antipsychotic and its main metabolites were determined at 6 weeks after starting the treatment to check adherence. Concentrations were measured by a validated high-performance liquid chromatography, using UV diode array detection and solid-phase extraction on cyano cartridges. Compounds were separated on a C8 reversed-phase column with a gradient of acetonitrile and phosphate buffer 50 mM, pH 3.8 and detected at 210 and 278 nm. To assure high precision and accuracy of the method, an internal standard was included in the sample preparation and quantitative evaluation. The within- and between-day precision expressed as coefficient of variation (CV)% was<10%. The quality of analyses was subject to a programme of internal quality control and external quality assessment 36 . The dose used for each patient was recorded, and results were standardized among different antipsychotic agents by converting the values to their equivalent olanzapine dose 37 ). Doses were titrated from week 0 to 4 and maintained thereafter for the duration of the study.

### Clinical parameters

Single appraisal of response and side effects might be confounded by different starting situations, therefore all outcome measures were computed as the difference between the value at the moment of treatment evaluation, that happened after 12 weeks, and the values at the start of the treatment. Response was assessed with the Positive and Negative Symptoms Scale (PANSS) 38 . While a 20% improvement in the PANSS score is often considered a threshold for treatment response, there is no real consensus about what percentage would be appropriate 39 . Therefore, the difference in PANSS from baseline to 12 weeks was considered as a continuous variable for the statistical analyses, which also provides a quantitative insight into the magnitude of the improvement. Adverse events were tracked with the Udvalg for Kliniske Undersøgelser (UKU) side effects rating scale 40 at the same time points. PANSS is a common way to assess the general severity of the illness across its two main axes (positive and negative symptoms) as well as a third dimension representing general psychopathology. Each axis is rated from 1 to 7, with higher scores indicating more severe symptoms. The three values can be added together to get a summary score. UKUs are a less widespread method of assessing side effects of psychotropic medications. It works by assessing a number of items representing different kinds of adverse effects, and each one is scored from 0 (representing absence) to 3 (representing greatest severity). In this study, the UKU scores were grouped into four categories: psychological, neurologic, autonomic and other side effects. A fifth and final category, total UKU, captures the overall emergence of any side effect, as it combines the scores of all the UKU categories.

### Polymorphism selection and genotyping

A candidate gene approach was used. A selection of glutamate genes previously associated with schizophrenia and/or treatment response (see introduction) were included in the study: GRIN1, GRIN2A, GRIN2B, GRIN3A, GRIA1, GRIK2, GRM2, GRM3, GRM5, GRM8, SLC1A1, SLC1A3 and GAD1. Forty-five TagSNPs within the candidate genes were selected using the ENSEMBL Genome Browser (www.ensembl.org), SNPinfo (snpinfo.niehs.nih.gov) and LDlink (ldlink.nci.nih.gov) using the parameters MAF≥0.05 and linkage of R ^2^ ≥0.8. A complete list of the genes and their polymorphisms is detailed in Table 2 . The SNPs have been chosen for their ability to capture genetic variability within the specified genes. Whole blood samples were taken from all study participants. DNA was extracted with a commercial kit (QIAmp DNA Mini Kit, Qiagen) following the manufacturers’ recommendations. Genotyping was performed with a MassARRAY platform (CEGEN-PRB2-ISCIII, University of Santiago de Compostela, Spain).

**Table:** **Table 2** Genetic polymorphisms included in the study.

Gene	Polymorphism	Gene	Polymorphism
GAD1	rs2058725	GRIN3A	rs1004362
GAD1	rs769407	GRIN3A	rs1323423
GRIA1	rs10515697	GRIN3A	rs1323427
GRIA1	rs1864205	GRIN3A	rs1983812
GRIA1	rs1994862	GRIN3A	rs2050639
GRIK2	rs2227281	GRIN3A	rs2050641
GRIK2	rs2518224	GRIN3A	rs2417290
GRIK2	rs2518302	GRM2	rs60972621
GRIK2	rs513216	GRM2	rs75938458
GRIK2	rs9404130	GRM3	rs1468412
GRIK2	rs995640	GRM3	rs1989796
GRIN1	rs2301364	GRM3	rs6465084
GRIN1	rs34547970	GRM5	rs10501686
GRIN1	rs4880215	GRM5	rs1391873
GRIN1	rs71387806	GRM5	rs2648640
GRIN2A	rs10518151	GRM5	rs4628675
GRIN2A	rs10870198	GRM5	rs596370
GRIN2A	rs6497524	GRM8	rs1008907
GRIN2A	rs9931155	GRM8	rs1419489
GRIN2B	rs11055624	GRM8	rs2299516
GRIN2B	rs12582848	SLC1A1	rs1471786
GRIN2B	rs1806201	SLC1A3	rs13154397
GRIN2B	rs219934		

### Statistical analysis

Linear regression analyses were performed for quantitative phenotypes (differences in PANSS, and total, psychological, neurologic, autonomic and other UKUs). A model consisting of gender, age, dose (olanzapine equivalent) and smoking habits (number of daily cigarettes) as covariates and the genotype of the selected polymorphisms as predictor variables was used for the analyses. P-values were corrected for multiple comparisons using the False Discovery Rate method. All analyses were performed using PLINK 1.07 (Shaun Purcell, May 10th, 2010). Separate analyses were conducted for the total sample, including all treatments (ALL cohort, N=258), for the subgroup of patients treated with clozapine (CLOZ cohort, N=114) and the patients using drugs other than clozapine (OTHER cohort, N=144).

## Results

Five samples were excluded after quality control analyses for having less than 95% SNP and/or sample successful call rate. All genotyped polymorphisms were in Hardy-Weinberg equilibrium.

### Genetic associations with treatment response

Associations were observed between treatment response and polymorphisms in GRIK2 (rs995640 [p=0.05] and rs2227281 [p=0.05]), GRIN2A (rs9931155 [p=0.02] and rs6497524 [p=0.04]) and GRIN2B (rs11055624 [p=0.05] and rs12582848 [p=0.05]) when considering all patients. Within the CLOZ group, improvement in PANSS was associated with SLC1A3 rs13154397 [p=0.02], GRIK2 rs995640 [p=0.03] and rs2227281 [p=0.03] variants, and with GRIN3A rs2050639 [p=0.03] and GRIN2A rs6497524 [p=0.04] variants. No association was detected between the genetic variants investigated and efficacy when analysing the OTHER group. None of the previously reported results remained significant after correction for multiple comparisons (adjusted p>0.05 in all instances). These results are presented in Table 3 .

**Table:** **Table 3** Association of SNPs and haplotypes with PANSS.

		ALL ^1^		CLOZ ^2^		OTHER ^3^	
GENE	SNP	BETA	P ^4^	BETA	P ^4^	BETA	P ^4^
GAD1	rs2058725	2.42	0.18	6.73	0.08	1.07	0.60
GAD1	rs769407	− 2.43	0.18	− 5.05	0.14	− 0.77	0.72
GRIA1	rs10515697	0.95	0.59	0.19	0.96	1.80	0.35
GRIA1	rs1864205	0.57	0.74	2.84	0.49	− 0.72	0.70
GRIA1	rs1994862	1.25	0.48	0.22	0.96	2.05	0.27
GRIK2	rs2227281	− 3.30	**0.05**	− 9.23	**0.03**	− 1.93	0.28
GRIK2	rs2518224	− 2.43	0.38	− 3.70	0.65	− 1.86	0.51
GRIK2	rs2518302	0.24	0.90	1.84	0.72	− 0.59	0.77
GRIK2	rs513216	− 0.87	0.59	− 0.94	0.80	− 0.97	0.58
GRIK2	rs9404130	2.18	0.48	− 5.36	0.54	4.28	0.17
GRIK2	rs995640	− 3.36	**0.05**	− 9.60	**0.03**	− 2.03	0.26
GRIN1	rs2301364	0.45	0.84	− 2.97	0.47	2.21	0.40
GRIN1	rs34547970	1.21	0.65	− 1.01	0.83	2.87	0.38
GRIN1	rs4880215	− 1.31	0.43	− 0.66	0.86	− 0.81	0.66
GRIN1	rs71387806	− 1.33	0.68	− 0.92	0.89	− 2.24	0.54
GRIN2A	rs10518151	2.83	0.34	− 11.17	0.12	5.93	0.06
GRIN2A	rs10870198	− 1.71	0.33	− 3.31	0.42	− 1.03	0.58
GRIN2A	rs6497524	− 3.07	**0.04**	− 6.25	**0.04**	− 2.09	0.22
GRIN2A	rs9931155	− 3.40	**0.02**	− 4.83	0.10	− 2.57	0.13
GRIN2B	rs11055624	3.39	**0.05**	4.93	0.15	2.06	0.29
GRIN2B	rs12582848	− 3.31	**0.05**	− 2.30	0.51	− 3.10	0.10
GRIN2B	rs1806201	2.15	0.41	4.27	0.49	1.31	0.63
GRIN2B	rs219934	0.68	0.68	2.30	0.49	1.27	0.50
GRIN3A	rs1004362	1.04	0.62	− 1.09	0.86	0.65	0.77
GRIN3A	rs1323423	2.81	0.17	5.39	0.30	1.24	0.57
GRIN3A	rs1323427	− 3.51	0.06	− 6.86	0.14	− 0.77	0.71
GRIN3A	rs1983812	− 1.44	0.36	− 4.10	0.33	− 2.57	0.11
GRIN3A	rs2050639	− 2.35	0.17	− 9.19	**0.03**	0.97	0.61
GRIN3A	rs2050641	− 2.56	0.18	− 6.19	0.15	0.73	0.74
GRIN3A	rs2417290	1.36	0.45	− 3.99	0.40	1.77	0.36
GRM2	rs60972621	− 0.42	0.79	− 5.92	0.06	2.81	0.13
GRM2	rs75938458	− 0.45	0.92	− 15.07	0.18	4.60	0.32
GRM3	rs1468412	− 0.10	0.96	− 2.01	0.68	0.14	0.94
GRM3	rs1989796	2.02	0.21	0.59	0.88	2.60	0.12
GRM3	rs6465084	− 0.32	0.88	− 7.13	0.20	1.30	0.54
GRM5	rs10501686	1.02	0.52	0.16	0.97	1.83	0.29
GRM5	rs1391873	1.44	0.39	− 0.48	0.87	3.14	0.12
GRM5	rs2648640	− 0.55	0.76	5.77	0.14	− 2.73	0.17
GRM5	rs4628675	1.41	0.40	1.23	0.75	2.20	0.23
GRM5	rs596370	− 1.02	0.55	3.33	0.42	− 2.42	0.19
GRM8	rs1008907	− 0.54	0.72	− 1.15	0.70	− 0.41	0.82
GRM8	rs1419489	− 0.54	0.73	− 2.52	0.42	0.34	0.85
GRM8	rs2299516	1.35	0.38	0.65	0.85	1.79	0.31
SLC1A1	rs1471786	− 0.56	0.80	− 1.69	0.65	0.63	0.81
SLC1A3	rs13154397	1.88	0.21	6.62	**0.02**	− 0.19	0.91

1 (ALL) – group of all the study participants, 2 (CLOZ) – group of participants using clozapine, 3 (OTHER) – group of participants not using clozapine, 4 (P) – uncorrected p-value. Bold indicates a p-value≤0.05. SNPs: single nucleotide polymorphisms; PANSS: Positive and Negative Symptoms Scale

### Genetic associations with side effects

Variants in several genes (GRIN3A, GRIK2, GRM8, GRIN2A, SLC1A3, GRM5, GRIA1, GRM2 and GAD1) showed associations with adverse effects. A nominal association was observed between GRIN3A rs1004362 [p=0.03] and total adverse effects in the ALL cohort, but not in any of the subgroups. An association with GRIN3A rs2417290 [p=0.04] was found only in the OTHER subgroup . The GRIN3A rs1004362 variant was also found to be associated with psychologic side effects in the ALL [p=0.02] and CLOZ [p=0.03] groups, and was associated with other UKUs in the CLOZ subgroup [p=0.02]. Other polymorphisms in GRIN3A also showed association with different kinds of adverse -effects only in the CLOZ subgroup (rs1323423 [p=0.02] for psychologic UKUs, rs2050641 [p=0.02] and rs1323427 [p=0.03] for autonomic UKUs). Genetic polymorphisms in GRIK2 were also related to side effects, as rs995640 [p=0.03] and rs9404130 [p=0.03] were associated with total side effects in the CLOZ sample, but this effect was not seen in the ALL or OTHER groups. The GRIK2 rs995640 variant was found to be associated with autonomic side effects in the patients of the ALL [p=0.02] and OTHER [p=0.03] subgroups, but not in the CLOZ sample. Another polymorphism in GRIK2, rs2518224, was associated with other adverse effects only in the CLOZ sample [p=0.04]. The GRM8, rs2299516 variant showed association with total adverse effects only in the CLOZ sample [p=0.04]. This polymorphism along with rs1008907 in the same gene were also associated with psychologic side effects only in patients of the CLOZ subgroup [p=0.03]. A third polymorphism in GRM8, rs1419489, showed association with other adverse effects in the CLOZ sample [p=0.03] as well as in the ALL sample [p=0.05]. Two polymorphisms in GRIN2A (rs6497524 and rs9931155) were associated with psychologic side effects in ALL [rs6497524 p=0.01, rs9931155 p=0.03] and OTHER patients [rs6497524 p=0.01, rs9931155 p=0.01]. GRIN2A rs6497524 was also associated with autonomic side effects in OTHER patients [p=0.01], but this was not detected in any of the other two subgroups. The SLC1A3 rs13154397 polymorphism was associated with neurologic adverse effects in the ALL [p=0.01] and OTHER samples [p=0.03] The GRM5 rs4628675 polymorphism was associated with neurologic side effects in the ALL group [p=0.05], and the polymorphism rs2648640 in the same gene was associated with other adverse effects in the OTHER subgroup [p=0.03]. Finally, the GRIA1 rs1864205 polymorphism was associated with autonomic side effects in the ALL and CLOZ samples [p<0.01 for both groups], and the association in CLOZ remained borderline significant after correction for multiple comparisons (p=0.05). Two other polymorphisms showed association with autonomic UKUs GRM2 rsr60972621 in the ALL sample [p=0.02] and GAD1 rs2058725, in the OTHER subgroup [p=0.05]. After correction for multiple comparisons, all adjusted p-values were higher than 0.05, except for the association between GRIA1 rs1864205 and autonomic side effects that bordered the significance threshold (adjusted p=0.05). A summary of these results can be seen in Table 4 .

**Table:** **Table 4** Association of SNPs and polymorphisms with UKUs

		UKUT ^1^						UKUP ^2^					
		ALL ^6^		CLOZ ^7^		OTHER ^8^		ALL ^6^		CLOZ ^7^		OTHER ^8^	
GENE	SNP	BETA	P ^9^	BETA	P ^9^	BETA	P ^9^	BETA	P ^9^	BETA	P ^9^	BETA	P ^9^
GAD1	rs2058725	0.78	0.34	1.17	0.52	0.79	0.38	0.03	0.95	0.43	0.68	0.05	0.93
GAD1	rs769407	–1.34	0.09	–1.59	0.31	–1.08	0.23	–0.54	0.23	–1.06	0.24	–0.31	0.55
GRIA1	rs10515697	–0.34	0.67	0.62	0.73	–0.28	0.75	–0.34	0.47	1.23	0.25	–0.64	0.21
GRIA1	rs1864205	0.16	0.84	2.11	0.22	–0.71	0.42	–0.08	0.87	0.23	0.82	–0.31	0.53
GRIA1	rs1994862	–0.19	0.81	0.61	0.74	–0.10	0.91	–0.35	0.44	1.23	0.25	–0.67	0.18
GRIK2	rs2227281	–0.37	0.63	–3.39	0.06	0.57	0.51	–0.11	0.81	–1.62	0.13	0.36	0.46
GRIK2	rs2518224	1.09	0.39	–1.55	0.67	1.20	0.37	0.86	0.23	1.97	0.36	0.60	0.43
GRIK2	rs2518302	–0.73	0.42	–2.68	0.18	–0.02	0.99	0.26	0.61	–1.23	0.30	0.77	0.16
GRIK2	rs513216	0.72	0.33	–0.89	0.57	1.27	0.13	0.12	0.77	–1.29	0.16	0.55	0.23
GRIK2	rs9404130	0.95	0.50	7.69	**0.03**	0.11	0.94	–0.52	0.52	3.40	0.11	–0.91	0.28
GRIK2	rs995640	–0.97	0.22	–3.83	**0.03**	–0.30	0.73	–0.35	0.44	–2.07	**0.05**	0.04	0.94
GRIN1	rs2301364	–0.08	0.94	–0.75	0.69	0.28	0.81	0.97	0.08	0.49	0.65	1.15	0.07
GRIN1	rs34547970	0.49	0.68	–0.73	0.74	1.19	0.40	1.07	0.11	0.44	0.72	1.35	0.09
GRIN1	rs4880215	0.33	0.66	0.51	0.77	0.54	0.50	0.22	0.59	–0.32	0.74	0.62	0.17
GRIN1	rs71387806	0.51	0.72	2.69	0.38	–0.45	0.77	0.38	0.64	0.93	0.59	<0.01	1.00
GRIN2A	rs10518151	0.14	0.91	–1.82	0.62	0.51	0.71	1.18	0.12	0.75	0.72	1.31	0.09
GRIN2A	rs10870198	0.06	0.93	0.07	0.97	0.17	0.84	–0.22	0.61	–1.42	0.18	0.14	0.76
GRIN2A	rs6497524	–1.07	0.12	–0.10	0.95	–1.22	0.10	–0.98	**0.01**	–0.43	0.60	–1.15	**0.01**
GRIN2A	rs9931155	–0.60	0.36	1.00	0.46	–0.73	0.32	–0.78	**0.03**	0.44	0.58	–1.09	**0.01**
GRIN2B	rs11055624	0.83	0.27	0.63	0.69	0.71	0.39	0.54	0.21	1.53	0.08	0.05	0.91
GRIN2B	rs12582848	–0.53	0.47	0.65	0.68	–0.76	0.35	–0.17	0.67	0.58	0.52	–0.32	0.49
GRIN2B	rs1806201	0.12	0.87	–1.31	0.65	0.51	0.73	0.47	0.54	–0.18	0.91	0.39	0.66
GRIN2B	rs219934	0.72	0.60	1.95	0.19	0.67	0.41	0.07	0.86	1.29	0.13	0.41	0.38
GRIN3A	rs1004362	–2.10	**0.03**	–4.16	0.08	–1.82	0.08	–1.25	**0.02**	–2.90	**0.03**	–1.05	0.07
GRIN3A	rs1323423	–1.02	0.27	–3.18	0.12	–0.52	0.62	–0.77	0.14	–2.77	**0.02**	–0.33	0.57
GRIN3A	rs1323427	–0.85	0.32	–2.46	0.20	0.31	0.76	–0.41	0.40	–0.50	0.66	0.05	0.93
GRIN3A	rs1983812	0.52	0.48	–3.00	0.08	0.09	0.91	0.34	0.40	–1.54	0.13	0.16	0.72
GRIN3A	rs2050639	–0.69	0.38	–0.77	0.68	0.00	1.00	–0.18	0.70	0.46	0.67	0.08	0.87
GRIN3A	rs2050641	–0.56	0.51	–1.51	0.39	0.52	0.61	–0.35	0.48	–0.18	0.86	0.05	0.93
GRIN3A	rs2417290	–1.46	0.07	–0.84	0.66	–1.83	**0.04**	–0.69	0.14	–0.90	0.42	–0.79	0.12
GRM2	rs60972621	–0.48	0.50	–1.50	0.31	0.26	0.75	–0.09	0.82	0.11	0.90	–0.03	0.95
GRM2	rs75938458	–2.74	0.16	–8.17	0.26	–1.24	0.53	–0.01	0.99	1.70	0.68	0.30	0.78
GRM3	rs1468412	–1.37	0.12	–1.58	0.43	–1.27	0.19	–0.28	0.57	–1.40	0.23	–0.03	0.96
GRM3	rs1989796	0.92	0.21	1.52	0.38	0.76	0.35	0.52	0.22	1.45	0.15	0.33	0.47
GRM3	rs6465084	–1.51	0.11	–1.57	0.49	–1.56	0.13	–0.01	0.98	0.21	0.88	–0.12	0.84
GRM5	rs10501686	–0.23	0.74	–1.73	0.29	0.62	0.40	–0.25	0.53	–1.06	0.25	0.18	0.67
GRM5	rs1391873	–0.19	0.80	0.65	0.64	–0.93	0.29	–0.30	0.47	–0.38	0.63	–0.42	0.40
GRM5	rs2648640	0.52	0.51	2.19	0.22	0.05	0.96	0.00	0.99	1.15	0.26	–0.39	0.43
GRM5	rs4628675	–0.04	0.96	–1.69	0.34	1.00	0.20	–0.25	0.55	–1.08	0.29	0.24	0.59
GRM5	rs596370	0.34	0.66	2.74	0.14	–0.31	0.70	0.16	0.70	1.33	0.21	–0.15	0.73
GRM8	rs1008907	–0.21	0.75	–1.67	0.21	–0.01	0.99	0.10	0.79	–1.47	**0.05**	0.57	0.20
GRM8	rs1419489	–0.65	0.35	–2.52	0.07	–0.17	0.83	0.09	0.82	–1.24	0.12	0.47	0.29
GRM8	rs2299516	0.92	0.18	3.07	**0.04**	0.39	0.60	–0.01	0.99	2.02	**0.02**	–0.53	0.21
SLC1A1	rs1471786	–0.50	0.61	–0.92	0.59	–0.09	0.94	<0.01	1.00	–0.46	0.64	0.44	0.50
SLC1A3	rs13154397	0.21	0.75	0.67	0.60	–0.15	0.84	–0.13	0.72	–0.21	0.78	–0.21	0.62

1 (UKUT) – Total UKU, 2 (UKUP) – Psychological UKU, 3 (UKUN) – Neurologic UKU, 4 (UKUA) – Automonic UKU, (5) UKUO – Other UKU, 6 (ALL) – group of all the study participants, 7 (CLOZ) – group of participants using clozapine, 8 (OTHER) – group of participants not using clozapine, 9 (P) – uncorrected p-value. Bold indicates a p-value≤0.05. UKUs: Udvalg for Kliniske Undersøgelser; SNPs: Single nucleotide polymorphisms

**Table:** **Table 4** Continued

		UKUN ^3^						UKUA ^4^					
		ALL ^6^		CLOZ ^7^		OTHER ^8^		ALL ^6^		CLOZ ^7^		OTHER ^8^	
GENE	SNP	BETA	P ^9^	BETA	P ^9^	BETA	P ^9^	BETA	P ^9^	BETA	P ^9^	BETA	P ^9^
GAD1	rs2058725	0.06	0.84	0.51	0.42	–0.23	0.53	0.46	0.09	0.71	0.37	0.49	**0.05**
GAD1	rs769407	–0.10	0.75	0.04	0.94	0.00	1.00	–0.21	0.43	0.24	0.72	–0.41	0.10
GRIA1	rs10515697	0.22	0.49	0.31	0.60	0.38	0.32	–0.16	0.55	–0.39	0.61	–0.08	0.76
GRIA1	rs1864205	–0.03	0.94	0.25	0.67	–0.08	0.82	0.74	**<0.01**	2.26	**<0.01**	0.13	0.60
GRIA1	rs1994862	0.21	0.50	0.31	0.61	0.36	0.33	–0.16	0.52	–0.38	0.63	–0.09	0.71
GRIK2	rs2227281	0.19	0.54	–0.42	0.50	0.42	0.24	–0.37	0.14	–0.88	0.26	–0.28	0.23
GRIK2	rs2518224	0.82	0.10	0.59	0.62	0.82	0.14	–0.36	0.37	–1.88	0.22	–0.32	0.38
GRIK2	rs2518302	–0.54	0.12	–0.63	0.35	–0.50	0.22	–0.22	0.44	–0.53	0.55	–0.09	0.75
GRIK2	rs513216	0.37	0.19	–0.05	0.92	0.59	0.08	0.25	0.30	0.44	0.51	0.12	0.60
GRIK2	rs9404130	0.54	0.33	0.78	0.52	0.48	0.44	–0.20	0.66	2.81	0.07	–0.65	0.11
GRIK2	rs995640	0.04	0.90	–0.41	0.50	0.20	0.59	–0.58	**0.02**	–1.26	0.10	–0.51	**0.03**
GRIN1	rs2301364	–0.41	0.27	–0.21	0.75	–0.51	0.27	–0.62	0.06	–1.22	0.13	–0.26	0.41
GRIN1	rs34547970	–0.45	0.32	–0.29	0.70	–0.51	0.36	–0.34	0.39	–1.03	0.28	0.09	0.81
GRIN1	rs4880215	0.21	0.46	0.43	0.48	0.10	0.75	0.11	0.65	0.41	0.58	0.09	0.70
GRIN1	rs71387806	0.20	0.71	0.54	0.61	0.18	0.78	0.40	0.40	0.68	0.61	0.17	0.70
GRIN2A	rs10518151	–0.14	0.79	–0.28	0.83	–0.20	0.73	0.02	0.96	–1.36	0.39	0.30	0.44
GRIN2A	rs10870198	0.29	0.33	0.61	0.35	0.19	0.56	0.11	0.67	0.39	0.63	0.06	0.78
GRIN2A	rs6497524	0.16	0.54	–0.26	0.60	0.41	0.18	–0.35	0.12	0.18	0.77	–0.49	**0.01**
GRIN2A	rs9931155	0.15	0.54	–0.17	0.72	0.43	0.14	–0.16	0.47	0.28	0.64	–0.23	0.26
GRIN2B	rs11055624	–0.03	0.92	–0.54	0.32	0.14	0.69	0.12	0.64	0.10	0.89	0.03	0.90
GRIN2B	rs12582848	–0.28	0.31	0.29	0.60	–0.44	0.18	–0.23	0.36	–0.55	0.42	–0.06	0.80
GRIN2B	rs1806201	0.28	0.55	–0.04	0.96	0.20	0.72	–0.36	0.49	–1.53	0.24	–0.27	0.59
GRIN2B	rs219934	0.18	0.51	0.64	0.22	0.50	0.13	–0.06	0.81	0.23	0.73	–0.07	0.76
GRIN3A	rs1004362	0.22	0.55	0.01	0.99	0.28	0.52	–0.34	0.27	0.17	0.87	–0.49	0.08
GRIN3A	rs1323423	0.43	0.23	0.49	0.48	0.41	0.34	–0.12	0.69	0.14	0.88	–0.22	0.44
GRIN3A	rs1323427	–0.25	0.44	–0.55	0.39	0.06	0.89	–0.20	0.45	–1.75	**0.03**	0.29	0.27
GRIN3A	rs1983812	–0.13	0.65	–0.39	0.51	–0.17	0.60	0.37	0.11	–1.36	0.07	0.11	0.61
GRIN3A	rs2050639	–0.25	0.41	–0.33	0.58	0.02	0.95	–0.19	0.45	–1.37	0.08	0.14	0.58
GRIN3A	rs2050641	0.20	0.56	0.05	0.93	0.55	0.20	–0.38	0.17	–1.71	**0.02**	0.15	0.58
GRIN3A	rs2417290	0.18	0.58	0.33	0.60	0.13	0.73	–0.21	0.43	0.65	0.42	–0.48	**0.05**
GRM2	rs60972621	–0.06	0.82	–0.14	0.78	0.03	0.92	–0.55	**0.02**	–1.22	0.06	–0.22	0.32
GRM2	rs75938458	–0.87	0.22	–4.11	0.10	–0.61	0.43	–0.87	0.16	–5.63	0.07	–0.09	0.86
GRM3	rs1468412	–0.43	0.20	–0.10	0.87	–0.46	0.25	–0.08	0.77	0.28	0.74	–0.13	0.63
GRM3	rs1989796	0.33	0.25	0.53	0.36	0.30	0.37	–0.04	0.87	–0.70	0.35	0.09	0.69
GRM3	rs6465084	–0.62	0.10	–0.94	0.21	–0.53	0.22	–0.20	0.51	–0.88	0.37	–0.04	0.89
GRM5	rs10501686	0.48	0.07	0.77	0.17	0.44	0.15	–0.29	0.20	–1.26	0.07	0.12	0.56
GRM5	rs1391873	–0.10	0.73	0.33	0.49	–0.32	0.38	0.15	0.56	0.65	0.28	–0.25	0.30
GRM5	rs2648640	–0.19	0.53	–0.35	0.57	–0.08	0.81	0.44	0.10	1.46	0.06	0.16	0.51
GRM5	rs4628675	0.55	**0.05**	0.68	0.27	0.60	0.06	–0.29	0.23	–0.97	0.21	0.07	0.76
GRM5	rs596370	–0.15	0.60	–0.14	0.83	–0.14	0.66	0.22	0.37	1.28	0.12	–0.02	0.93
GRM8	rs1008907	–0.21	0.42	–0.27	0.57	–0.25	0.45	0.07	0.77	0.09	0.87	<0.01	0.98
GRM8	rs1419489	–0.22	0.40	–0.26	0.60	–0.20	0.54	–0.05	0.83	–0.14	0.82	–0.05	0.81
GRM8	rs2299516	0.32	0.21	0.69	0.20	0.24	0.43	0.30	0.18	0.37	0.58	0.25	0.24
SLC1A1	rs1471786	–0.35	0.34	–0.46	0.44	–0.31	0.52	0.18	0.58	0.11	0.88	0.23	0.47
SLC1A3	rs13154397	0.66	**0.01**	0.74	0.09	0.64	**0.03**	0.13	0.55	0.50	0.37	–0.07	0.75

**Table:** **Table 4** Continued

		UKUO ^5^					
		ALL ^6^		CLOZ ^7^		OTHER ^8^	
GENE	SNP	BETA	P ^9^	BETA	P ^9^	BETA	P ^9^
GAD1	rs2058725	0.32	0.25	–0.44	0.39	0.64	0.06
GAD1	rs769407	–0.32	0.24	–0.82	0.06	–0.11	0.75
GRIA1	rs10515697	–0.09	0.77	–0.44	0.43	–0.03	0.93
GRIA1	rs1864205	–0.43	0.13	–0.69	0.19	–0.35	0.31
GRIA1	rs1994862	0.15	0.61	–0.45	0.42	0.28	0.42
GRIK2	rs2227281	–0.14	0.62	–0.54	0.33	0.03	0.93
GRIK2	rs2518224	–0.25	0.59	–2.22	**0.04**	0.13	0.80
GRIK2	rs2518302	–0.08	0.82	–0.40	0.53	0.03	0.94
GRIK2	rs513216	–0.19	0.39	–0.01	0.99	–0.23	0.38
GRIK2	rs9404130	0.71	0.16	0.40	0.72	0.78	0.18
GRIK2	rs995640	–0.20	0.42	–0.15	0.79	–0.14	0.62
GRIN1	rs2301364	–0.16	0.62	0.18	0.74	–0.34	0.43
GRIN1	rs34547970	–0.03	0.94	0.18	0.77	–0.14	0.80
GRIN1	rs4880215	–0.24	0.34	–0.04	0.93	–0.28	0.36
GRIN1	rs71387806	–0.46	0.35	0.46	0.60	–0.72	0.23
GRIN2A	rs10518151	–0.35	0.44	–0.74	0.48	–0.24	0.65
GRIN2A	rs10870198	–0.06	0.81	0.38	0.47	–0.13	0.68
GRIN2A	rs6497524	–0.20	0.38	0.41	0.31	–0.40	0.16
GRIN2A	rs9931155	–0.07	0.74	0.48	0.21	–0.22	0.43
GRIN2B	rs11055624	0.15	0.57	–0.55	0.22	0.47	0.15
GRIN2B	rs12582848	0.02	0.95	0.31	0.49	–0.12	0.70
GRIN2B	rs1806201	0.43	0.26	0.52	0.43	0.29	0.57
GRIN2B	rs219934	0.03	0.91	–0.23	0.58	–0.04	0.89
GRIN3A	rs1004362	–0.50	0.14	–1.63	**0.02**	–0.20	0.63
GRIN3A	rs1323423	–0.33	0.32	–1.21	**0.05**	–0.01	0.98
GRIN3A	rs1323427	–0.06	0.85	0.49	0.41	–0.36	0.35
GRIN3A	rs1983812	0.01	0.96	0.31	0.57	0.11	0.71
GRIN3A	rs2050639	–0.11	0.70	0.59	0.30	–0.38	0.29
GRIN3A	rs2050641	0.06	0.84	0.37	0.50	–0.13	0.74
GRIN3A	rs2417290	–0.52	0.08	–1.05	0.07	–0.33	0.35
GRM2	rs60972621	–0.01	0.96	–0.26	0.54	0.15	0.63
GRM2	rs75938458	–0.67	0.29	1.86	0.37	–0.75	0.29
GRM3	rs1468412	–0.41	0.19	–0.28	0.65	–0.48	0.20
GRM3	rs1989796	0.22	0.41	0.31	0.57	0.17	0.59
GRM3	rs6465084	–0.48	0.16	0.24	0.73	–0.68	0.09
GRM5	rs10501686	–0.25	0.29	–0.15	0.75	–0.23	0.41
GRM5	rs1391873	–0.02	0.94	0.04	0.91	–0.06	0.87
GRM5	rs2648640	0.53	**0.05**	–0.08	0.87	0.69	**0.03**
GRM5	rs4628675	–0.14	0.57	–0.27	0.59	–0.05	0.88
GRM5	rs596370	0.28	0.29	0.25	0.64	0.26	0.40
GRM8	rs1008907	–0.15	0.52	–0.02	0.97	–0.35	0.25
GRM8	rs1419489	–0.47	**0.05**	–0.85	**0.03**	–0.43	0.15
GRM8	rs2299516	0.26	0.27	0.03	0.95	0.40	0.17
SLC1A1	rs1471786	–0.14	0.69	–0.15	0.76	–0.12	0.78
SLC1A3	rs13154397	–0.07	0.74	–0.35	0.33	0.03	0.91

## Discussion

We hypothesized that polymorphisms in glutamatergic genes might affect the outcomes of antipsychotic treatments in schizophrenia patients. This hypothesis was put to the test by analysing polymorphisms in genes related to glutamate pathways and comparing the results with standardized variables (PANSS and UKU) in a cohort of patients using antipsychotic medications. While statistically significant results regarding efficacy or side effects have been found for glutamatergic genes, studies up until now have been centred on particular drugs, particular side effects and/or particular genes. This study is unique in that it observes a wider range of treatment outcomes, such as efficacy and different classes of adverse effects.

We used a candidate-gene strategy to maximise the possibilities of finding associations of moderate effects that may be missed in genomic studies. The study revealed several trends for the association with polymorphisms in genes controlling glutamatergic neurotransmission. Most of the associations were found on the subgroup of patients treated with clozapine (CLOZ), particularly for efficacy. The associations of GRIK2 variants with PANSS, GRIA1 variants with autonomic UKUs and GRM8 variants with other UKUs were stronger in the CLOZ sample, as represented by the lower p-values observed in this cohort. Additionally, the association between the GRIA1 rs1864205 variants and autonomic side effects was statistically significant even after correcting for multiple comparisons (adjusted p value=0.05), but only in the CLOZ subgroup. An association for the same polymorphism was found in the total (ALL) cohort but it was no longer significant after FDR corrections. This suggests an effect on autonomic adverse drug reactions that would be specific to clozapine, or at least more relevant for clozapine than for other drugs. GRIK2, GRIA1, GRM8 and GRIN2A could be pharmacogenetic biomarkers for patients using clozapine, provided that these associations are replicated in future studies. GRIA1 expression has been reported to be reduced in animal models of hypertension 41 , providing a link between this receptor and autonomic phenotypes. GRIA1 genetic variants have also been associated with haloperidol efficacy 42 . While we did not find GRIA1 variants associated with treatment response, the patients in our study were treated mainly with second generation antipsychotics which may explain the discrepancy.

Our sample contains an abundance of treatment-resistant patients who have failed antipsychotic treatment in the past. Since clozapine is only used in patients with previous failure to antipsychotic medication, the CLOZ subgroup represents a group of patients with treatment-resistant schizophrenia. Clozapine also represents the final step in the prescription of antipsychotic drugs, because at this point, there are no more alternative drugs. The results of this study, therefore, are particularly relevant for the subset of the population where pharmacogenetic information that can improve the treatment is most needed. The identification of glutamatergic genes as possible factors influencing the improvement of PANSS and UKU scores is an encouraging step forward in the development of better tools for personalizing antipsychotic treatments, but this and the other findings in this study should be validated with bigger sample sizes. As expected, no gene variant was found to individually predict the success or failure of antipsychotic treatment. Given the multitarget profile of most antipsychotics, it is likely that the integration of different clinical and genetic variables is required to predict efficacy.

Our study has several limitations. Sample size was moderate, making it difficult to identify associations with variants of small effect or with rare polymorphisms. While the statistical power was deemed sufficient to detect large to moderate effects, small effects would require bigger sample sizes to be detected. Since drug response is a polygenic trait, small effect sizes from factors are, in fact, likely. Therefore, it is possible that some small effects remain undetected. In addition, the patients were treated with different antipsychotics, although the majority (96.1%) were second-generation drugs. Even though doses were standardized to their olanzapine equivalents, their different profiles regarding their affinity to neurotransmitter receptors and mechanism of action were potential confounders. The separate analysis of the CLOZ sample led us to overcome the heterogeneity issue, at the cost of a further reduced sample size. Some patients used lorazepam concurrently with their antipsychotic medication. This can lead to drug interactions like increased sedation. However, the use was sporadic and low doses (1–1.5 mg/day) were used. Furthermore, no increased sedation was observed in these patients. None of the results, except one, was statistically significant after multiple comparison corrections, and replication in independent samples are required to confirm their validity, as is the case in most candidate-gene studies. A heterogeneous range of side effects was included in the category of “Other” side-effects, which may have hampered the finding of specific associations. Finally, since non-Caucasian patients had to be excluded from the analyses due to the low numbers recruited, the findings of this study might not apply to other ethnicities.

In summary, this article provides evidence suggesting that genes affecting glutamatergic signalling, particularly GRIA1, might affect both efficacy and safety of antipsychotic treatments, and hints at a bigger effect in treatment-resistant patients, represented here by the CLOZ sample. Nevertheless, these findings should be replicated to confirm the association and to provide a better estimation of their clinical utility as response predictors.

## Financial support and sponsorship

This study was supported by grants from Institute Carlos III (FIS PI11/02006 and g FIS PI16/01029), ISCIII-SGEFI/FEDER

## Contributors

Study design: Marc Cendrós, Rosa Catalán, MD, María J Arranz, PhD

Molecular biology studies: Marc Cendrós, Mercè Torra, PhD, Mercè Brunet, PhD, , Alexandre Serra-Llovich, Marta H. Hernandez

Sample recruitment and clinical data obtention: Rosa Catalán, MD, Rafael Penadés, PhD, Alexandre González-Rodríguez, MD, PhD, Josefina Perez-Blanco, MD

Statistical analyses: Marc Cendrós, Rosa Catalán, MD, Natalia Cullell, PhD, María J Arranz, PhD

Manuscript draft: Marc Cendrós, Rosa Catalán, MD, Natalia Cullell, PhD, María J Arranz, PhD

All authors contributed to and have approved the final manuscript.
